# Cardiovascular Abnormalities in Juvenile Dermatomyositis: A Scoping Review for the Clinical Rheumatologists

**DOI:** 10.3389/fmed.2022.827539

**Published:** 2022-06-24

**Authors:** Sanjib Mondal, Prabal Barman, Pandiarajan Vignesh

**Affiliations:** Allergy Immunology Unit, Department of Pediatrics, Advanced Pediatrics Centre, Post Graduate Institute of Medical Education and Research, Chandigarh, India

**Keywords:** dermatomyositis, vasculopathy, cardiac dysfunction, acute, long-term, screening, imaging

## Abstract

Juvenile dermatomyositis (JDM) is a common form of inflammatory myositis in children. Vasculopathy and endothelial dysfunction play significant roles in the pathogenesis of JDM. Cardiac involvement in JDM is often underestimated, and it may be a potential indicator of poor prognosis. Cardiac dysfunction in JDM can occur both in the acute and chronic stages of the disease. Amongst the acute complications, acute congestive heart failure (CHF), myocarditis, arrhythmia, and complete heart block are common. However, these remain unrecognized due to a lack of overt clinical manifestations. Increased rates of cardiovascular abnormalities have been noted with anti-SRP and anti-Jo 1 auto-antibody positivity. Long-term follow-up studies in JDM have shown an increased prevalence of hypertension, atherosclerosis, coronary artery disease, and metabolic syndrome in adolescence and adulthood. Monitoring of body-mass index, blood pressure, and laboratory evaluation of fasting glucose and lipid profile may help in identifying metabolic syndrome in children with JDM. Steroid-sparing agents, daily exercise, and a healthy diet may reduce such long-term cardiac morbidities. Current use of multimodality imaging such as stress-echocardiography, contrast-enhanced echocardiography, cardiac magnetic resonance imaging, and positron emission tomography has increased the diagnostic yield of subclinical heart disease during acute and chronic stages of JDM. This review elaborates on different aspects of cardiac dysfunction in JDM. It also emphasizes the importance of cardiac screening in long-term follow-up of children with JDM.

## Introduction

Juvenile dermatomyositis (JDM) is a common form of inflammatory myositis in children (1). Characteristic clinical features include proximal muscle weakness associated with typical skin lesions, such as heliotrope rash over eyelids and Gottron papules (2). However, any central organ system can be involved in JDM due to systemic vasculopathy (3). Cardiac involvement is often overlooked considering its subclinical course, although it can often cause significant morbidity and mortality. Oppenheim first reported the involvement of the cardiovascular system in patients with dermatomyositis in Oppenheim (4). With the rapid advancement of imaging techniques and cardiac monitoring tools in recent decades, cardiac complications in JDM are increasingly being recognized.

The exact etiopathogenesis of JDM, especially cardiovascular involvement, has remained an enigma despite decades of research. Histopathology studies have highlighted that systemic vasculopathy plays an integral part in the pathogenesis of JDM (5). Elevated interferon (IFN) signature in JDM and systemic vasculopathy could result in endothelial dysfunction (6). Endothelial damage leads to the progression of vasculopathy. Chronic endothelial dysfunction and systemic inflammation can also predispose patients with JDM to accelerated atherosclerosis (7, 8). In recent years, the significance of type I IFNs in the context of the pathogenesis of JDM has been extensively studied. Several studies have revealed the presence of a distinguishing gene signature suggestive of a type I IFN pathway activation (IFNα/β) in peripheral blood and muscle tissues of children with JDM (9). It has also been observed that these gene signatures strongly correlate with disease activity, suggesting their role as potential biomarkers in JDM. Moneta et al. even reported that expression levels of IFNγ, IFNγ-inducible genes (type II IFN score), and tumor-necrosis factor (TNFα) were significantly high in untreated patients with JDM, suggesting the role of type II IFN as well (9).

**FIGURE 1:**
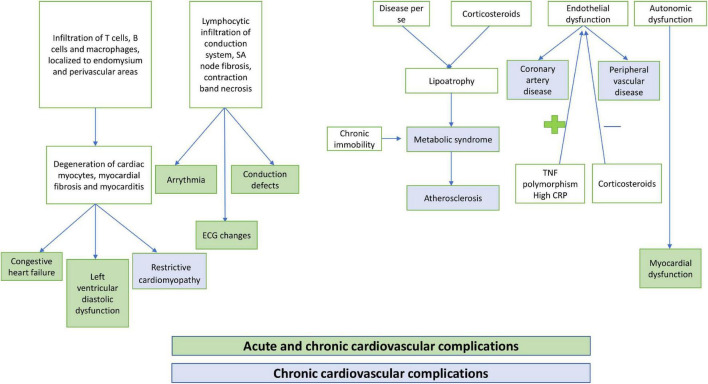
Pathogenesis of cardiovascular complications in children with juvenile dermatomyositis (JDM).

Cardiac dysfunction in JDM may either present acutely or manifest late during the disease course. Among the acute complications, congestive heart failure (CHF), myocarditis, arrhythmia, and complete heart block have been commonly noted (Figure 1). However, these remain unrecognized in most instances due to a lack of overt clinical manifestations. Late cardiovascular complications are also relatively common in JDM. Left ventricular diastolic and systolic dysfunction, hypertension, atherosclerosis, coronary artery disease, and metabolic syndrome are frequently encountered in adolescence and adulthood. Dyslipidemia, hypertension, abdominal obesity, and impaired glucose tolerance, either due to chronic disease processes or as a complication of long-term use of corticosteroid therapy, may also affect cardiovascular health in children with JDM. Chronic immobility and autonomic dysfunction also contribute to cardiovascular changes in JDM (10, 11). Different ethnic backgrounds, sedentary lifestyles, and dietary habits also impact the prevalence of metabolic syndrome in JDM (12).

There are no consensus guidelines for screening and earlier detection of silent heart disease in children with JDM. Newer imaging modalities such as tissue Doppler imaging, cardiac scintigraphy, and stress echocardiography have also identified subclinical cardiovascular abnormalities in children with JDM. Regular monitoring of carotid media intima thickness and brachial artery reactivity index in children with JDM has been reported to predict the future development of atherosclerosis (13). Clinical assessment such as the extent of lipoatrophy, blood pressure monitoring, and waist circumference measurement can also aid in the early identification of metabolic syndrome in JDM.

Thus, the etiology of cardiac morbidities in JDM is multifactorial and judicious screening, and monitoring of risk factors may reduce overall cardiac morbidity and mortality in children with JDM.

## Search Criteria for Articles

A comprehensive search was made using the Web of Science, Scopus, and PubMed databases to gather English articles published from 1980 to 2021 on cardiovascular abnormalities in JDM. We incorporated the following words in the search strategy: “Juvenile dermatomyositis,” “vasculopathy of JDM,” “cardiac dysfunction in JDM,” “acute and long-term complications in JDM,” “screening methods in JDM,” “newer imaging modalities in JDM,” and “management of cardiac dysfunction in JDM (Table 1).”

**TABLE 1:** Previous studies on cardiovascular complications in children with JDM.

Author, country, year	Age (years)	Sex (male:female)	Number of JDM patients	Follow-up (months)	Cardiac abnormalities described	Remarks
Pachman et al., United States, (18)	1.75–13.75	3:4	21	NA	*Acute*: ECG abnormalities, left ventricular dysfunction *Chronic:* NA	ECGs were abnormal in 13 patients, with left ventricular hypertrophy noted in 5
Rider et al., United States, Canada and Europe, (97)	4–9.25	31:63	94	18	*Acute:* Hypertension, ventricular dysfunction *Chronic:* NA	Myositis damage index was described to validate to outcome in JDM with cardiovascular system being included in one of four domains
Na et al., South Korea, (17)	4–12	3:5	16	3–110	ECG abnormalities: ST-T changes, right bundle branch block	ECG abnormalities were found in 6 out of 16 patients with JDM (37.5%)
Schwartz et al., Norway, (20)	1.4–17.3	23:36	59	NA	ECG abnormalities: poor R-wave progression, left ventricular hypertrophy signs, right bundle branch block, pathological Q-wave, P pulmonale and prolonged QTc Echocardiographic changes: Left ventricular dysfunction, valvular regurgitation Hypertension	JDM patients had subclinical left ventricular diastolic dysfunction Patients with elevated E/E’ (early diastolic transmitral flow/early diastolic tissue velocity) also had high prevalence of pathological ECG and hypertension High disease activity 1-year post diagnosis predicted high E/E’ at follow-up
Lu et al., China, (76)	16–50*	15:31*	46*	NA	*Acute*: Left ventricular diastolic dysfunction	Tissue Doppler imaging was useful in detecting early cardiac complications like left ventricular diastolic dysfunction in patients with dermatomyositis
Huber et al., United States, (19)	5.1–11.6	NA	329	51.6	Congestive cardiac failure	Factors associated with mortality in their cohort of juvenile idiopathic inflammatory myopathies Of 8 deaths in JDM patients, 1 was due to congestive cardiac failure
Barth et al., Norway, (11)	NA	21:34	55	162	Arrhythmia (Heart rate variability) Systolic/diastolic dysfunction	Heart rate variability is decreased in patients with JDM compared with controls. Lower heart rate variability is associated with systolic and diastolic cardiac dysfunction and high-sensitivity CRP and active disease.
Cantez et al., Canada, (15)	2–17.6	11:24	105	122.4	ECG changes (*n* = 69): Prolonged QTc, prolonged PR and wide QRS Echocardiographic changes (n = 52): pericardial effusions, tricuspid regurgitation, pulmonary insufficiency, aortic insufficiency, mitral valve prolapse, mitral regurgitation, increased right ventricular end diastolic dimension and decreased pulmonary vein D-wave velocity (left ventricular ejection fraction was normal in all)	Cardiac abnormalities at disease onset are frequently seen, but are rarely significant findings. JDM patients should be considered for screening for cardiac disease as late cardiac complications are well recognized.
Diniz et al., Brazil, (35)	11.9–13.3	11:24	35	NA	LV systolic dysfunction Myocardial dysfunction	LV two-dimensional speckle-tracking echocardiography can detect early systolic myocardial compromise in asymptomatic patients with preserved EF. Longitudinal strain impairment was associated with disease activity and cumulative damage, whereas circumferential strain impairment was associated exclusively with cumulative damage.

*Lu et al. included both polymyositis and dermatomyositis in their cohort. NA, not available; ECG, electrocardiogram; JDM, juvenile dermatomyositis; CRP, C-reactive protein; LV, left ventricle; EF, ejection fraction.

## Prevalence of Cardiovascular Abnormalities

The exact prevalence of cardiac abnormalities in JDM is unknown, and various authors have reported it to vary from 9 to 72% (14). Cantez et al., in a retrospective study on 105 patients with JDM from Canada, noted abnormalities in electrocardiogram (ECG) and echocardiography in 6 and 25% of children (15). A multicentric study from Latin America and Europe reported cardiac involvement in 2.9% of the 490 patients with JDM (16). In a Korean cohort, the authors found that 6 out of 14 children had changes in ECG (17). Pachman et al. reported abnormalities in ECG in 65% (13/20) of children with JDM in their cohort (18). As can be interpreted above, there is a wide variation in the prevalence of cardiac morbidity in children with JDM and likely causes may include lack of screening due to subtle clinical features as well as the absence of advanced imaging modalities at most centers and hitherto unknown genetic differences across different population groups. Nevertheless, cardiac disease was a significant cause of mortality in JDM in certain studies. A study noted three deaths out of 17 deaths (*n* = 329) with cardiac dysfunction; however, these patients also had other comorbidities (19).

## Acute Cardiac Complications in Children With Juvenile Dermatomyositis

Studies have reported a high prevalence of electrocardiographic findings (6% had ECG changes; 25% had echocardiographic changes) (15) and subclinical ventricular diastolic dysfunction in children with JDM (20). The abnormal ECG findings commonly noted include atrial/ventricular arrhythmia, bundle branch block, AV block, abnormal Q waves, and prolongation of PR interval (20). Singh et al. reported a case of ventricular arrhythmia from their cohort of 33 children with JDM (21). Bradycardia has also been reported in a child with JDM (22).

An autopsy study of 16 patients with dermatomyositis showed evidence of myocarditis (25%), focal myocardial fibrosis (25%), and coronary artery changes in 5 patients (31%) (medial sclerosis with calcification and intimal proliferation in 1 each; active vasculitis in 3) (23). Banker et al. performed an autopsy on eight patients with JDM and reported the presence of inflammatory cells in the sub-endocardium and myocardium; however, none of them had symptomatic heart disease (24).

Pericardial involvement is relatively rare in children with JDM compared to myocardial involvement. However, as a part of systemic vasculopathy and inflammation, pericarditis may be observed in a subset of children with JDM. One study has reported pericarditis in 12–25% of patients with JDM (20). A multicentric study from Europe on 18 children with juvenile idiopathic inflammatory myositis (JIIM) reported that 50% of their children with positive anti-PL7 antibodies had pericarditis during their disease course (25). Pericardial tamponade has also been reported in a patient with JDM (26).

Autonomic dysfunction has been reported to occur in certain patients with dermatomyositis (27). One study showed reduced heart rate variability in patients with JDM due to autonomic instability, and such patients also had myocardial dysfunction (28). Barth et al. demonstrated a correlation between reduced heart rate variability and cardiac dysfunction. Furthermore, the authors noted a positive correlation with high serum levels of inflammatory cytokines, such as TNF alpha, IL-6, monocyte chemoattractant protein-1 (MCP-1) and eotaxin, and cardiac dysfunction (11).

### Myositis-Specific Antibodies and Cardiac Dysfunction

A high prevalence of ECG abnormalities in children with JIIM with positive anti-SRP antibodies has been noted (29). Similarly, in a study on the correlation between clinical and myositis specific/myositis-associated antibodies in a subset of patients with adult-onset myositis, overt cardiac manifestations (arrhythmia, cardiomyopathy, and fibrosis) were observed in all four patients with positive anti-SRP antibody (30). A European cohort of patients with inflammatory myositis reported a 4.15-fold increased risk of cardiac involvement in the presence of anti-SRP antibody positivity (31). Albayda et al. showed cardiac involvement (myocarditis, arrhythmia, and cardiomyopathy) in patients with IIM (*n* = 6/7) with anti-mitochondrial antibody positivity (32). A 13-year male child with JDM had severe cardiomyopathy in the presence of a positive anti-MDA5 antibody (33). However, a Dutch study showed no cardiac morbidity in their cohort of 5 patients with positive anti-SRP antibodies (34). Studies from large and multicentric cohorts are needed to understand the spectrum of cardiovascular abnormalities amongst different auto-antibody subgroups in JDM.

## Delayed Onset Cardiac Diseases in Children With Juvenile Dermatomyositis

Cardiac morbidities are not uncommon during long-term follow-up of children with JDM. However, there is a lack of data on this aspect. With corticosteroid therapy, mortality in children with JDM has reduced significantly in past decades. The improved survival rate has led to increased recognition of chronic morbidities, including cardiovascular complications in children with JDM. The pathogenesis for long-term cardiac complications is different from that of acute cardiovascular complications in JDM. While many patients with JDM have subclinical heart disease, a subset of them can present with overt cardiac manifestations secondary to metabolic syndrome during adolescence and adulthood.

Schwartz et al. demonstrated diastolic dysfunction in 22% of children with JDM at a median duration of 16.8 years after initial diagnosis. The authors noted a correlation between myositis damage index (MDI) score, skin disease activity at 1 year, disease duration, and cardiovascular dysfunction. The authors also postulated that myocardial remodeling in JDM was an ongoing chronic process (20). Diastolic dysfunction appears earlier when compared to systolic dysfunction, and systolic dysfunction seems to be less frequent than diastolic dysfunction (28). Diniz et al. detected systolic myocardial compromise (reduction in left ventricular longitudinal and circumferential strain) in 35 asymptomatic children with JDM with preserved ejection fraction by 2-dimensional speckle tracking echocardiography (35). A comparative study on the 6-min walking test (6MWT) among 23 adults with inflammatory myositis and 18 healthy age-matched controls showed reduced distance covered by patients with myositis due to reduced stroke volume and interstitial lung disease. However, there were no patients with JDM in their cohort, and disease duration was not specified (36).

In JDM, metabolic syndrome is a chronic complication that leads to future cardiovascular compromise in children during adolescence or adulthood. Metabolic syndrome constellates dyslipidemia, hypertension, truncal obesity, and impaired glucose tolerance (37). Lipoatrophy in patients with JDM and prolonged use of corticosteroids for disease control play a role in the development of metabolic syndrome in children with JDM (38). Interestingly, cardiac dysfunction has been noted with metabolic syndrome even in the absence of overt heart disease. Heart rate turbulence, a measure of vagal activity, can predict the risk of cardiovascular disease in metabolic syndrome (39). Verma et al. demonstrated lipoatrophy in 65% of patients with JDM from their cohort (*n* = 20), and 66% had hypertriglyceridemia at a mean follow-up period of 2.2 years. However, none of them had glucose intolerance (40). Silverberg et al. showed a higher incidence of hypertension (OR 22.25), obesity (OR 5.87), diabetes mellitus (OR 7.95), dyslipidemia (OR 5.84), lipodystrophy (OR 151.08), and organ and peripheral atherosclerosis (10.09) in children with JDM when compared with age-matched healthy controls (41). A study from the National Institute of Health (NIH) has also shown a high rate of cardiovascular risk factors in children with JDM (*n* = 17, mean duration of active disease course 38 months; 70% had either polycyclic or chronic active disease), such as abnormal lipid profile (47%), hypertension (23%), and obesity (47%) (42).

Coyle et al. showed that beta-cell function was not affected in patients with JDM. However, they reported impaired glucose tolerance test (35.2%), elevated fasting insulin (41.2%), high glucose-insulin ratio (47.1%), and elevated insulin resistance (47.1%) in 17 patients with myositis (16 children with JDM with a mean of 38 months of active disease). Furthermore, they described a positive correlation between muscle inflammation and elevated glucose-insulin ratio, high blood sugar level, and a negative correlation between cytokine profile (increased IL2 and IL12; decreased IL1RA and IL10) and serum glucose/glucose-insulin ratio (42).

In a study with adult patients with a history of JDM, authors reported high carotid intimal thickness and brachial artery reactivity index despite them being young and having lower body mass index (43) (Supplementary Material). Weng et al. showed a twofold higher risk for the development of coronary heart disease in children with JDM compared to healthy controls (44). Angina pectoris/Prinz metal angina has been reported in adults with dermatomyositis due to coronary vasospasm (45). A study has shown that Raynaud’s phenomenon increased the chance of coronary vasospasm (46). One child with JDM was reported to have coronary artery dilatation which was an unusual finding (47).

Hypertension has been reported in 20% of patients with JDM (20). Hypertension in JDM may be due to metabolic syndrome, premature atherosclerosis, and chronic use of glucocorticoids. Silverberg et al. reported a higher risk of hypertension (OR 40.85) in patients with JDM between 1 and 9 years than in healthy controls (41). Authors also reported a higher risk of arrhythmia (OR 3.77) and obesity (OR 20.20) in this cohort compared with age-matched normal children. In addition, they showed a higher risk of obesity in Whites when compared with Hispanics and Blacks. However, hypertension was more common amongst Asians, and diabetes mellitus was more common amongst Blacks. Besides, the Black population had a high risk of developing bradycardia and cerebrovascular disease (41). A study by Mendez et al. corroborates that hypertension was noted more amongst the Asian population, although the incidence of JDM was high amongst Caucasians (48). This suggests that different genetic predispositions, lifestyle patterns, and differences in access to healthcare facilities may also impact patients with JDM who develop metabolic syndrome. Few studies have reported physical inactivity as a crucial risk factor for the development of cardiovascular disease in several rheumatological conditions, including JDM (49).

Cardiovascular disease in JDM also has a significant impact on mortality. A study from Hungary showed that cardiovascular involvement was the main reason for mortality in 55% of cases in JIIM (50). A Norwegian study reported 14% deaths due to cardiac disease in their cohort of juvenile idiopathic myositis (51). Silverberg et al. showed an increased risk of cerebrovascular morbidity in children with JDM, such as cerebral infarct (OR 10.82), cerebrovascular disease (OR 15.49), and transient ischemic attack (OR 10.82) (41).

## Cardiovascular Involvement Due to Drugs Used in Juvenile Dermatomyositis

Certain drugs, such as glucocorticoids and hydroxychloroquine (HCQ), that are commonly used in the management of JDM, have also been associated with cardiac dysfunction (52, 53). There is an increased risk of cardiovascular disease with the use of glucocorticoids, depending on the dose and duration of treatment. Thus, patients on JDM with long-term steroid treatment should have a cardiovascular risk prevention plan considering the time and amount of steroid exposure (52) (Supplementary Material).

Although cardiac complications with HCQ are rare, they can occasionally result in irreversible damage and death. The two main clinical manifestations reported are conduction abnormalities (bundle or atrioventricular block) and myocardial hypertrophy (53).

Recently, Janus kinase (JAK) inhibitors have also been tried in refractory cases of JDM. However, most published case reports have used JAK inhibitors in the context of refractory cutaneous disease and arthritis, especially in adults (54–56). A case series by Kurasawa et al. has reported the successful use of tofacitinib in interstitial lung disease in anti-MDA5 antibody-positive dermatomyositis in adults (57). The use of ruxolitinib in the pediatric-age group was first reported by Aeschlimann et al. in a 13-year-old girl with refractory JDM (58). Another case report by Papadopoulou et al. reported the successful use of baricitinib in an 11.5-year-old boy with refractory JDM (59). Although the literature suggests that JAK inhibitors can increase the risk of dyslipidemia and thromboembolism in adults (60), long-term studies are needed to assess the cardiovascular side effects of these drugs in JDM.

Other immunosuppressants, such as methotrexate and mycophenolate mofetil, may have a protective effect on the heart due to a reduction in overall inflammatory burden (Supplementary Table 3) (52, 53, 60–67).

## Risk Factor Assessment, Disease Monitoring, and Detection of Silent Heart Disease

There are no consensus guidelines about screening intervals and monitoring for prediction and earlier detection of cardiovascular disease in JDM. However, thorough clinical examination and essential laboratory evaluations may yield clues to recognize cardiovascular complications in JDM (Supplementary Material).

Nail-fold capillaroscopy (NFC) is usually used for disease activity monitoring by reading end row capillary loops on nail folds. A previous study has shown a positive correlation between NFC changes and skin disease in JDM (68). A study showed a correlation between NFC changes with lung disease and not with cardiac involvement in patients with JDM (69).

An echocardiogram may detect subclinical heart disease both at diagnosis and on follow-up. ECG detects several underlying silent conduction defects. Screening echocardiography is also essential for earlier recognition of myocardial dysfunction, pericarditis, and valvular heart disease. An image-guided myocardial biopsy can detect subtle myocardial inflammation; however, it is difficult to perform routinely.

There are no available biomarkers to detect underlying heart disease in patients with JDM. However, one study showed a positive correlation between high serum levels of TNF alpha, IL-6, and eotaxin with cardiac dysfunction (11). Type 1 IFNs regulate various cytokines and proteins associated with disease activity in JDM. It has been found that elevated neopterin, CXCL10, CXCL11, and galectin-9 levels correlate with disease activity in JDM. It has been suggested that these parameters may act as potential biomarkers to assess disease activity in JDM (9). Pro-brain natriuretic peptide (BNP) is raised in CHF; however, it has not been studied in JDM. Creatinine kinase (CK): MB isotype (CKMB) is usually elevated in any myocardial damage (70). However, it is not specific to myocardial dysfunction in children with JDM as these children also have inflammatory changes in skeletal muscles (70). Troponin is another biomarker for myocardial damage. Several isotypes are commercially available, such as cardiac troponin I (cTnI) and troponin T (cTnT). cTnT was noted to be elevated in patients with inflammatory myositis without cardiac disease (71). One study with 39 adult patients with IIM showed elevated cTnT in 19 patients (41%), while only one had elevated cTnI (70). cTnT shows cross-reactivity with skeletal muscle TnT due to structural homology. However, different amino acid sequences (31 different amino acids) of cTnI do not show any cross-reactivity with skeletal TnT. So, cTnI may be useful for the earlier detection of silent heart diseases in patients with inflammatory myositis (71, 72).

Abdominal obesity can be assessed by measuring waist circumference (73). However, it cannot differentiate between subcutaneous fat tissue and visceral abdominal fat. A study from Iran has shown that high amounts of visceral fat correlate with increased carotid intimal media thickness (CMT), a surrogate marker for atherosclerosis (74). Dual-energy X-ray absorptiometry (DEXA) and magnetic resonance imaging (MRI) can accurately detect fat distribution in internal organs (75). Measurement of subcutaneous fat tissue by slide calipers is another helpful tool for assessing lipodystrophy in children with JDM (40). Regular check-up of blood pressure is also vital for earlier identification of hypertension. Baseline investigations such as lipid profile, random blood sugar, and glycosylated hemoglobin are useful ancillary investigations for diagnosing metabolic syndrome.

In the last few decades, rapid advances in radiodiagnosis have increased the detection rate of silent cardiac dysfunction in children with JDM. Conventional echocardiography may occasionally miss systolic dysfunction with preserved ejection fraction; however, it has been observed that with the use of 2-dimensional speckle tracking echocardiography, mild systolic dysfunction can also be picked up (35). Tissue Doppler imaging is another helpful modality for detecting cardiac morbidity in patients with JDM especially left ventricular dysfunction (76). MRI and myocardial scintigraphy can detect myocardial perfusion (77, 78). Flow-mediated dilatation and CMT are helpful for the earlier detection of atherosclerosis (13).

Intravascular ultrasound and optical coherence tomography are essential tools for detecting coronary changes and changes in large peripheral vessels (79). Coronary calcium score in computed tomography (CT) angiography can predict the internal status of coronaries (80). Perivascular lipid volume, which regulates vascular stiffness, can be identified by multidetector CT and MRI (81). However, the utility of these imaging modalities has not been studied in patients with JDM or DM. Furthermore, such sophisticated tools and techniques are not available at most centers.

### Role of Cardiac Magnetic Resonance Imaging in Juvenile Dermatomyositis

Cardiac MRI is a valuable non-invasive tool to assess myocardial inflammation and scarring. A study on cardiac MRI on 16 adults with inflammatory myositis (9 DM and 7 PM) showed epicardial and intramyocardial late gadolinium enhancement (LGE) in 56.3% of the patients suggesting active inflammation. LGE was more commonly noted amongst patients with PM when compared to DM (*p* = 0.060) (82). Another study by Rosenbohm et al. on 53 patients with inflammatory myositis (64.2% PM, 24.5% DM, and 7.5% non-specific myositis) showed that patients with reduced left ventricular ejection fraction had an increased incidence of LGE. Notably, 54% of the patients with DM had LGE, and on most occasions, the lateral segment was involved compared to the anterior segment or septum (77).

### Role of Cardiac Positron Emission Tomography Imaging in Juvenile Dermatomyositis

Since Gould et al. first described the utility of cardiac Positron emission tomography (PET) in clinical practice, its use in various inflammatory and autoimmune diseases has seen rapid strides. The premise for using [18F] Fluorodeoxyglucose PET (FDG-PET) is that FDG accumulates in inflammatory lesions where glucose-consuming inflammatory cells are present. Studies in adults with polymyositis and dermatomyositis have shown that inflammatory lesions, especially cardiac lesions, can be identified by calculating the maximum standardized uptake value (SUVmax). However, there are no such studies in children with JDM so far. Somatostatin receptor-targeted PET imaging with 68Ga-DOTANOC (68Ga-DOTA-NaI-octreotide) or 68Ga-DOTATOC (68Ga-DOTA-D-Phe-Tyr-octreotide) (Gallium-based tracers) can have better results in the detection of active cardiac lesions by eliminating the effects of glucose uptake by normal cardiomyocytes. Cardiac PET imaging may have a prominent role in the future, especially in identifying cardiovascular abnormalities in JDM (83, 84).

## Management of Cardiovascular Disease in Children With Juvenile Dermatomyositis

Due to multifactorial etiopathogenesis, therapy with immunosuppressants alone may not be sufficient to prevent cardiovascular morbidity in JDM. There is a lack of literature on cardiac complications in children with JDM. Studies in adult patients with dermatomyositis have shown that cardiac dysfunction can develop even while on steroid therapy (85). Also, adult literature has shown no correlation between cardiac disease and active skeletal muscle inflammation. Some of these patients with dermatomyositis had developed cardiac dysfunction even without active skeletal muscle inflammation (86).

The Childhood Arthritis and Rheumatology Research Alliance (CARRA) and Single hub and access point for pediatric rheumatology in Europe (SHARE) have recommended the use of intravenous immunoglobulin (IVIg) in the context of moderate to severe disease activity, especially severe cutaneous disease in JDM (87, 88). However, it is pertinent to note that since IVIg has an anti-inflammatory and immunomodulatory effect, it may enhance the clearance of inflammatory cytokines that contribute to damage of cardiac myocytes and may have a role in managing active myocarditis. IVIg has been used in many centers for managing active myocarditis in children irrespective of the underlying etiology (89, 90). However, further studies would be required to assess whether IVIg can supplant other immunosuppressive drugs in the management of myocarditis in JDM.

The long-term management is mainly centered around earlier identification of metabolic syndrome and prevention of the development of complications such as coronary artery disease. Supportive care for CHF, arrhythmia, and hypertension is required. It is imperative to screen for metabolic abnormalities during follow-up of children with JDM.

Diet modification in metabolic syndrome is another factor that can lead to a favorable outcome. A calorie-restricted diet may be recommended to prevent complications due to metabolic syndrome (91). Physical exercise in JDM improves muscle strength in JDM and improves aerobic capacity, thereby improving cardiac health. de Oliveira et al. showed that exercise training improves B cell function and metabolic parameters and attenuates cardiovascular risks in patients with autoimmune myopathies (92).

Initiation of hypolipidemic drugs, such as statins and fibrates and lifestyle modifications, may be warranted in some patients to control dyslipidemia and prevent premature atherosclerosis (93). However, the use of statins in the setting of inflammatory myositis is not without controversy. Statins have been linked to the development of autoimmune necrotising myopathy (94, 95). The International myositis assessment and clinical study (IMACS) group evaluated the use of statins in 1,641 patients with myositis. A total of 36 out of 300 patients with inflammatory myositis who received lipid-lowering therapies had worsening myopathy, and the majority of them had exposure to statins. Further research is required to address whether statins can be safely recommended for the management of hyperlipidemia in patients with inflammatory myositis (96).

## Conclusion

Cardiac complications in children with JDM are not uncommon. Cardiac disease constitutes a significant cause of mortality in children with JDM. However, these are under-recognized on most occasions due to a lack of overt manifestations. A sizeable proportion of children with JDM develop cardiac dysfunction over time. Regular screening and monitoring of risk factors should be carried out for earlier diagnosis and better outcomes. Initiation of lifestyle modification and a balanced diet may significantly reduce cardiac morbidities due to metabolic complications. Besides ECG and conventional echocardiography, several sophisticated imaging tools have evolved over the last few decades, and they may be instrumental in detecting silent heart disease. In addition, timely transition to adult healthcare providers is also essential for earlier detection and management of cardiovascular complications and eventual better outcomes.

## Author Contributions

SM and PB: preparation of the first draft of the manuscript and literature review. PV: inception of the idea, critical review of the manuscript, and final approval. All authors contributed to the article and approved the submitted version.

## Conflict of Interest

The authors declare that the research was conducted in the absence of any commercial or financial relationships that could be construed as a potential conflict of interest.

## Publisher’s Note

All claims expressed in this article are solely those of the authors and do not necessarily represent those of their affiliated organizations, or those of the publisher, the editors and the reviewers. Any product that may be evaluated in this article, or claim that may be made by its manufacturer, is not guaranteed or endorsed by the publisher.
