# Supplementary material for: Cardiovascular Abnormalities in Juvenile Dermatomyositis: A Scoping Review for the Clinical Rheumatologists
Source: Front Med (Lausanne). 2022 Jun 24;9:827539. doi: 10.3389/fmed.2022.827539 (PMC9263083; doi:10.3389/fmed.2022.827539)
Supplement: Supplementary file 1 [file Table_1.DOCX]

**Supplementary table 1: Clinical monitoring and laboratory tools for cardiac dysfunction in JDM**

| **Clinical parameters** | **Biomarkers** | **Imaging tools** | **Histopathology** | **Others** |
| --- | --- | --- | --- | --- |
| - Body weight, waist circumference, body mass index (BMI) - Quantification of lipoatrophy (slide callipers) - Blood pressure | - Blood sugar/HbA_1_C - Serum lipid profile - Serum troponin (cTnI) - Creatinine kinase MB isotype (CKMB) - Serum Pro brain natriuretic peptide - Serum cytokines (Galectin, Eotaxin, monocyte chemoattractant protein) | - Echocardiogram (ECG) - Echocardiography - Tissue doppler imaging - 2-dimensional speckle tracking echocardiography - Cardiac magnetic resonance imaging (contrast enhanced) - Cardiac scintigraphy (99m_TC_-PYP) - CT angiography for coronary calcification - Flow mediated dilatation - Carotid media intimal thickness (CMIT) | - Image guided myocardial biopsy | - ? Nail-fold capillaroscopy (NFC) |

**Supplementary table 2: Comparison of cardiac manifestations of juvenile dermatomyositis and adult-onset myositis**

| Parameters | Adult-onset myositis | Juvenile dermatomyositis |
| --- | --- | --- |
| Incidence | Incidence of cardiac involvement varies amongst different studies (6-75%). Higher detection of subclinical cardiac involvement may be more due to frequent use of echocardiogram in triage amongst adult populations. | Exact incidence of cardiac involvement in JDM is not known, probably it is underestimated. Furthermore, lack of use of invasive diagnostic tests underestimates the exact incidence. |
| Type of myositis | Cardiac involvement is more common with patients with polymyositis (PM) when compared to dermatomyositis. Actual estimate of cardiac health in inclusion body myositis (IBM) and autoimmune necrotizing myopathy (ANM) is unknown. | In children, cardiac involvement with myositis are mostly reported amongst patients with JDM probably due to predominant incidence of JDM in comparison to PM, IBM and ANM in adults. |
| Cardiac manifestations | Congestive heart failure is most common overt heart manifestations while arrythmia such as hemiblock, bundle branch block, atrial fibrillation is more common amongst subclinical cardiac manifestations. Detection of arrythmia in adults are more in comparison to children probably due to frequent use of ECG and presence of comorbidities in adult age. | Similarly, in children arrythmia is commonest subclinical manifestation, however, it is under recognized many times due to infrequent use of ECG at diagnosis and follow up. |
| Ventricular dysfunction | Ventricular diastolic dysfunction and systolic dysfunction was found more with prolonged disease duration, MSA/MAA positivity. | Ventricular dysfunction in JDM have been correlated with prolonged disease duration and skin disease activity at 1 year following disease onset. |
| Pulmonary artery hypertension (PAH) | PAH is more frequent in PM probably due to interstitial lung disease and associated malignancy in addition to isolated cardiac involvement. | Incidence of PAH in JDM is not known. |
| Restrictive cardiomyopathy | More common in adult-onset myositis. | Rarely reported in pediatric population. |
| Coronary artery disease | Probably more common in adult and earlier manifestation during disease course. | Coronary artery disease has been reported in case reports. Actual incidence is not known. |
| Association with MSA/MAA | Few reports have described relation between cardiac manifestations and anti SRP antibody and anti-mitochondrial antibody positivity.  **Anti SRP antibody**   - Rider et al (50%) - Targoff et al (33%) - Betteridge et al (2.4%)   **Anti-mitochondrial antibody**   - Albayda et al (71%) | No such correlation was established in pediatric age group. |
| Management | Treated with immunosuppressants like glucocorticoids and cyclophosphamide. Rituximab and cardiac transplantation have been carried out. | Most affected children have been treated with glucocorticoids and cyclophosphamide. |
| Outcome/Mortality | Mortality rate varies amongst different studies. 5-48% mortality rate have been reported due to cardiac cause in adult-onset myositis. | Exact burden is not known. One study reported 3 deaths due to cardiac causes amongst 329 patients with JDM (6). |

**Supplementary table 3: Cardiovascular effects of drugs used in JDM**

| **Drug** | **Use in JDM** | **Cardiovascular effect** | **Remark** |
| --- | --- | --- | --- |
| Glucocorticoid (52) | Mild/Moderate/Severe disease | - Hyperglycaemia - Hypertension - Dyslipidaemia - Central obesity - Metabolic syndrome - Coronary heart disease - Myocardial infarction - Heart failure - Stroke - Atrial fibrillation | There is an increased risk of cardiovascular disease with glucocorticoid use and it is dependent on dose and duration of treatment. All patients on JDM with long-term steroid treatment should have a cardiovascular risk prevention plan taking into account current and prior steroid use. |
| Methotrexate (92) | Mild/Moderate/Severe disease | - Anti-atherogenic effect | There is a reduced risk of cardiovascular disease with methotrexate use in the long run. However, rarely acute cardiac complications like arrythmias have been reported with high doses. |
| Hydroxychloroquine (HCQ) (53) | Cutaneous disease | - Conduction disorders - Ventricular hypertrophy - Hypokinesia - Heart failure - Pulmonary arterial hypertension - Valvular dysfunction | Although HCQ cardiac disorder is rare, it remains a potential cause of irreversible damage and death. The two main clinical manifestations reported are conduction disorders (bundle or atrioventricular block) and myocardial hypertrophy. |
| Cyclophosphamide (93) | Severe disease | - Tachyarrhythmias - Hypotension - Heart failure - Myocarditis - Pericardial disease | Cardiac complications of cyclophosphamide depend on dose and they usually present acutely. |
| Mycophenolate mofetil (94) | Severe disease | - Anti-atherogenic effect | There is a reduced risk of cardiovascular disease with mycophenolate mofetil use in the long run. |
| Azathioprine (95) | Severe disease | - Hypotension - Cardiogenic shock - Atrial fibrillation - Tachycardia | Cardiac complications are very rarely described with azathioprine, and mainly in the context of hypersensitivity reactions. |
| Cyclosporin (96) | Severe disease | - Hypertension - Tachycardia | With chronic use of cyclosporin, the possibility of hypertension increases and consequent increase in the risk of stroke, myocardial infarction, heart failure and other adverse cardiovascular events associated with elevated blood pressure. |
| Anti-tumour necrosis factor agents (Infliximab, adalimumab) (97,98) | Severe disease | - Reduced cardiovascular events - Rarely heart failure | Anti-tumour necrosis factor agents have a good safety profile with respect to cardiovascular complications. Very rarely, heart failure has been reported. |
| Janus kinase (JAK) inhibitors (60) | Severe disease | - Dyslipidaemia - Thromboembolism - Stroke | There is a risk of dyslipidaemia and thromboembolism, however, JAK inhibitors have been used rarely in children. Long term studies will be required before a definitive conclusion can be drawn. |
